# Case Report: CD19 CAR T-cell therapy following autologous stem cell transplantation: a successful treatment for R/R CD20-negative transformed follicular lymphoma with *TP53* mutation

**DOI:** 10.3389/fimmu.2023.1307242

**Published:** 2023-12-08

**Authors:** Jinjing Zhang, Dali Cai, Ran Gao, Yuan Miao, Yan Cui, Zhenghua Liu, Heyang Zhang, Xiaojing Yan, Nan Su

**Affiliations:** ^1^ Department of Hematology, The First Affiliated Hospital of China Medical University, Shenyang, Liaoning, China; ^2^ Department of Pathology, The First Affiliated Hospital of China Medical University, Shenyang, Liaoning, China; ^3^ Department of Nuclear Medicine, The First Affiliated Hospital of China Medical University, Shenyang, Liaoning, China

**Keywords:** transformed follicular lymphoma, diffuse large B-cell lymphoma, TP53 mutation, chimeric antigen receptor T-cell, autologous stem cell transplantation

## Abstract

**Background:**

Follicular lymphoma (FL), a common indolent B-cell lymphoma, has the potential to transform into an aggressive lymphoma, such as diffuse large B-cell lymphoma (DLBCL). The outcome of patients with transformed follicular lymphoma (tFL) is poor, especially in patients with transformed lymphoma after chemotherapy and patients with progression within 24 months (POD24). Chimeric antigen receptor (CAR) T-cell therapy combined with autologous stem cell transplantation (ASCT) has promising antitumor efficacy.

**Case presentation:**

Here, we described a 39-year-old male patient who was initially diagnosed with FL that transformed into DLBCL with POD24, CD20 negativity, *TP53* mutation, and a bulky mass after 3 lines of therapy, all of which were adverse prognostic factors. We applied a combination approach: CD19 CAR T-cell infusion following ASCT. Ibrutinib was administered continuously to enhance efficacy, DHAP was administered as a salvage chemotherapy, and ICE was administered as a bridging regimen. The patient underwent BEAM conditioning on days -7~ -1, a total of 3.8 × 10^6/^kg CD34^+^ stem cells were infused on days 01~02, and a total of 10^8^ CAR T cells (relmacabtagene autoleucel, relma-cel, JWCAR029) were infused on day 03. The patient experienced grade 2 cytokine release syndrome (CRS), manifesting as fever and hypotension according to institutional standards. There was no immune effector cell-associated neurotoxicity syndrome (ICANS) after CAR T-cell infusion. Finally, the patient achieved CMR at +1 month, which has been maintained without any other adverse effects.

**Conclusion:**

This case highlights the amazing efficacy of CD19 CAR T-cell therapy following ASCT for R/R tFL, thus providing new insight on therapeutic strategies for the future.

## Introduction

Follicular lymphoma (FL) is a common indolent B-cell lymphoma, and the expected overall survival potentially extends beyond 20 years (1). However, this disease is highly heterogeneous, and some FLs have the potential to transform into aggressive lymphomas; one such example of transformed follicular lymphoma (tFL) is diffuse large B-cell lymphoma (DLBCL) (2, 3). Transformed DLBCL has a lower complete remission (CR) rate and shorter progression-free survival (PFS) than *de novo* DLBCL after conventional chemotherapy (4). Progression of tFL within 24 months (POD24) and TP53 mutation are also strongly correlated with a poor outcome (3, 5, 6). There are no standard therapeutic approaches for tFL, and the outcome is poor, especially in those whose transformation occurs early after chemotherapy (2, 7).

Although high-dose chemotherapy combined with autologous stem cell transplantation (HDT-ASCT) is the standard strategy for refractory and relapse (R/R) DLBCL patients who achieve efficacy above partial remission, approximately 50% of patients will not benefit from such treatment (8–10). In recent years, chimeric antigen receptor (CAR) T-cell therapy has emerged as a promising treatment for R/R non-Hodgkin lymphoma (NHL) (11–14). However, the 1-year OS in *TP53*-altered LBCL was significantly lower than that in *TP53* wild-type LBCL (44% versus 76%, *P* =0.012) treated with CD19 CAR T cells (15). Moreover, primary resistance and relapse after CAR T-cell therapy remain major challenges. The efficacy of ASCT or CAR T-cell therapy alone still needs further improvement. A recent study found that for R/R B-NHL patients with *TP53* alterations treated with CAR19/22 T-cell therapy combined with ASCT, the estimated 2-year PFS and OS rates were 77.5% and 89.3%, respectively (5). This suggests that the combination of CAR T-cell therapy with ASCT is worthy of further clinical application due to its high effectiveness, good safety, and beneficial outcomes (5, 16, 17).

Here, we present a patient with R/R CD20-negative tFL with POD24, *TP53* mutation and a bulky mass. Complete metabolic remission (CMR) was achieved +1 month after autologous CD19 CAR T-cell infusion following ASCT, thus providing a meaningful combination treatment strategy for R/R tFL.

## Case presentation

A 39-year-old Chinese male was diagnosed with FL (grade 3b, stage III, group A, FLIPI was 3, high risk and FLIPI-2 was 2, intermediate risk) in January 2020. Immunohistochemical results for lymphoma cells of left cervical lymph nodes were as follows: BCL6 (+), MUM1 (+), Ki-67 (80%+), CD20 (+), Pax5 (+), BCL2 (+), CD10 (+), CyclinD1 (-), and sparsely positive for CD3, CD5, CD15, CD30, CD68, and C-MYC. EBER by *in situ* hybridization test was negative in the lymphoma cells. Multiple lymphadenopathies on both sides of the mediastinum with high uptake of 18F-fluorodeoxyglucose (FDG) were revealed by positron emission tomography (PET-CT). The patient obtained CMR by PET-CT evaluation after 3 cycles of R-CHOP (rituximab 375 mg/m^2^ day 0, cyclophosphamide 750 mg/m^2^ day 1, pirarubicin 50 mg/m^2^ day 1, vincristine 1.4 mg/m^2^ day 1, and prednisone 100 mg day 1-5). After receiving additional 3 cycles of R-CHOP and 1 cycle of maintenance R therapy, PET-CT indicated an enlarged lymph node with high uptake in the right pelvic cavity (SUVmax was 7, Deauville was 5), and the original disease relapsed early. As the patient had no symptoms at the time, he refused further biopsy and treatment.

**Figure 1:**
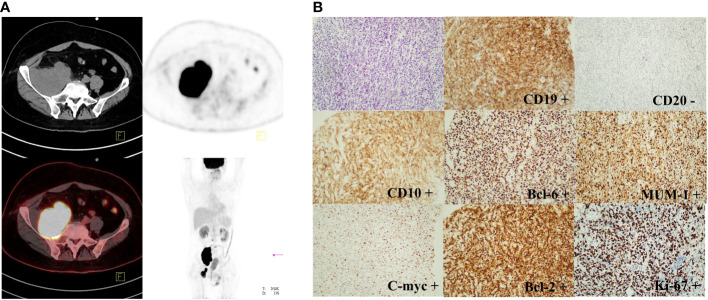
PET-CT scan and immunohistochemical staining results of FL transforming into DLBCL (GCB subtype). **(A)**: PET-CT imaging showed a bulky mass from the right psoas major muscle to the right pelvic wall (SUVmax was 42.1) and a soft tissue mass in the right groin (SUVmax was 21.2). **(B)**: The immunohistochemical staining results indicated that FL had transformed into DLBCL (GCB subtype) with CD20 negative, Bcl-6 (90%+), MUM1 (90%+), Ki-67 (80%+), Bcl-2 (90%+), c-myc (60%+), CD10 (+), and CD19 (+). (original magnification, 200×). The thin arrows represent the original comparable lesion locations.

In March 2021, the patient complained of pain in the back and lower extremities, and further core needle aspiration biopsy of the right inguinal lymph node revealed grade 2 FL. The patient received 1 cycle of BR (rituximab 375 mg/m^2^ day 0 and bendamustine 90 mg/m^2^ day 1,2). Although pain relief and thrombosis occurred in his right lower limb, the disease progressed with further enlargement of the soft tissue mass in the bilateral inguinal and right paravascular iliac arteries. Subsequently, 2 cycles of GB ( ortuzumab 1000 mg day 1, 8, 15 for the first cycle and day 1 for the second cycle, bendamustine 90 mg/m^2^ day 1, 2 for each cycle) were administered. Unfortunately, the disease showed no response to treatment with salvage immunochemotherapy. Then, PET-CT scans showed a bulky mass (a maximum diameter of 84 mm) from the right psoas major muscle to the right pelvic wall with increased FDG uptake (SUVmax was 42.1) and a soft tissue mass in the right groin with increased FDG uptake (SUVmax was 21.2) (**Figure 1A**). The third pathological investigation of the right paravascular iliac lesion indicated that the lymphoma had transformed into DLBCL (GCB subtype) that was negative for CD20 and positive for Bcl-6 (90%+), MUM1 (90%+), Ki-67 (80%+), Bcl-2 (90%+), c-myc (60%+), CD10 (+), and CD19(+) (**Figure 1B**). A negative rearrangement of MYC and a positive rearrangement of BCL2 were demonstrated by fluorescence *in situ* hybridization (FISH). Next-generation sequencing (NGS) of the paraffin-embedded lymphoma tissues was performed using the Illumina high-throughput sequencing platform technology. The results showed that *TP53* mutations were always present in 3 pathological specimens from different sites (**Table 1**). Moreover, there were more than 10 kinds of gene mutations, including *TP53*, *KMT2D*, *MS4A1*, *CD83*, *DUSP2*, *EZH2*, *FOXO1*, *HIST1H1C*, *HIST1H1E*, *NFKBIA*, *SGK1*, *SOCS1*, *STAT3*, and *TBL1XR1*.

**Table 1:** Gene mutations revealed by next-generation sequencing (NGS) of the paraffin-embedded lymphoma tissues in 3 pathological specimens of the present patient.

Gene	Variant sites (HGVS)	2020.1 FL grade 3b CD20 (+) CD19 (+)	2021.4 FL grade 2 CD20 (+) CD19 +)	2022.3 DLBCL (tFL) CD20 (-) CD19 (+)	COSMIC ID
*BOL7A*	NM _020993(BCL7A):c.92 + 1G> T		20.80%		COSV105069285
*BTG1*	NM_001731(BTG1): c.109_112delinsTTGT(p.Q38delinsX)	2.40%			
*CD83*	NM_001040280(CD83):c.38C>T (p.A13V)			1.22%	COSV105309260
*DUSP2*	NM_004418(DUSP2):c.433G> T (p.E145X)			1.00%	
*EZH2*	NM_001203247(EZH2):c.1922A> G (p.Y641C)			1.00%	COSV57446008
*FOXO1*	NM_002015(FOXO1):c.224G> C (p.S75T)			3.60%	COSV65427200
*HIST1H1B*	NM_005322(HIST1H1B):c.392C> T (p.A131V)		7.20%		COSV100137995
*HIST1H1C*	NM_005319(HIST1H1C):c.160delC(p.R54fs)			4.06%	
*HIST1H1C*	NM_005319(HIST1H1C):c.331G>A(p.A111T)	18.01%	11.09%	47.89%	COSV59190824
*HIST1H1C*	NM005319(HIST1H1C):c.417G>T(p.K139N)			20.53%	COSV59191946
*HIST1H1E*	NM_005321(HIST1H1E):c.358_367delinsACTAAAAAGT(p.A120_A123delinsTKKS)			12.09%	
*HIST1H1E*	NM_005321(HIST1H1E):c.535G>C (p.A179P)			13.26%	COSV104388611
*KMT2D*	NM_003482(KMT2D):c.11227C>T(p.Q3743X)	17.15%	14.38%	30.10%	
*KMT2D*	NM_003482(KMT2D):c.12667C>T(p.Q4223X)	18.58%	15.02%	31.94%	COSV105188200
*MS4A1*	NM_152866(MS4A1):c.160-1delG			54.33%	
*NFKBIA*	NM_020529(NFKBIA):c.202C>T(p.Q68X)			15.19%	COSV53751859
*NFKBIA*	NM_020529(NFKBIA):c205_208delinsTAGT(p.Q69_L70delinsX)			1.10%	
*NFKBIA*	NM_020529(NFKBIA):c.337-1G>C			1.03%	
*SGK1*	NM_001143676(SGK1):c.362C>G(p.A121G)			31.00%	COSV52811618
*SGK1*	NM_001143676(SGK1):c.385G>A(p.G129S)			11.09%	COSV105103899
*SGK1*	NM_001143676(SGK1):c.402T>G(p.I134M)			2.20%	COSV105104044
*SGK1*	NM_001143676(SGK1):c.402_423delinsCCAATAATTA(P.K136_S141delinsX)			25.82%	
*SGK1*	NM_001143676(SGK1):c.405_418delinsATAATT(p.K136X)			2.27%	
*SGK1*	NM_001143676(SGK1):c.563C>T(P.A188V)			1.64%	
*SGK1*	NM_001143676(SGK1):c.896_905delinsTTGAAATAGT(p.A299_A302delinsVEIV)			1.00%	
*SOCS1*	NM_003745(SOCS1):c.174C>G(p.F58L)				
*SOCS1*	NM_003745(SOCS1):c.174_175delinsGA(p.F58_R59delinsLS)		16.54%	30.74%	
*STAT3*	NM_003150(STAT3):c.1840A>C(p.S614R)		1.61%		COSV52888203
*TBL1XR1*	NM_024665(TBL1XR1):c.1044T>A(p.H348Q)	14.31%	12.06%	31.48%	COSV70504936
*TP53*	NM_000546(TP53):c.713G>A(p.C238Y)	21.42%	15.29%	46.35%	COSV52661646

% refers to the gene mutation frequency. (+), positive and (-), negative expression.

**Figure 2:**
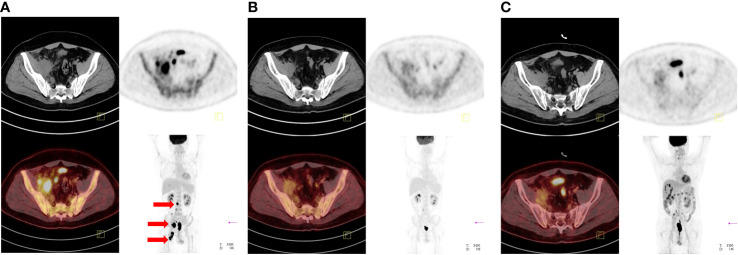
PET-CT evaluation before and after ASCT and CAR T-cell infusion. **(A)**: PET-CT examination demonstrated PD before ASCT and CAR T-cell infusion. **(B)**: PET-CT examination demonstrated CMR in the +1 month after ASCT and CAR T-cell therapy. **(C)**: PET-CT examination demonstrated sustained CMR in the +3 month after ASCT and CAR T-cell therapy. The thick arrows represent the positive lesions; thin arrows represent the original comparable lesion locations.

The patient received CD19 CAR T-cell infusion following ASCT therapy. Auto hematopoietic stem cells were collected after 1 cycle of DHAP (dexamethasone 40 mg day 1-4, cisplatin 100 mg/m^2^ day 1, cytarabine 2 g/m^2^ q12h day 2) with the BTK inhibitor ibrutinib (560 mg/day) administered continuously as salvage chemotherapy. One month later, autolymphocyte apheresis was performed to manufacture CAR T cells. During the production of CD19 CAR T cells, 1 cycle of ICE (ifosfamide 5000 mg/m^2^ day 2, carboplatin 737 mg day 2, etoposide 100 mg/m^2^ day 1-3) was administered as a bridging regimen. PET-CT evaluation before ASCT and CAR T-cell infusion indicated PD with a novel intraperitoneal tumor mass near the posterior abdominal wall and other remaining masses (**Figure 2A**). The patient underwent BEAM conditioning (carmustine 300 mg/m^2^ day -7, etoposide 200 mg/m^2^ day -6~-3, cytarabine 200 mg/m^2^ q12h day -6~-3, and melphalan 140 mg/m^2^ day -2) and ASCT following CD19 CAR T-cell infusion. On May 28, 2022 (day 01), and May 29, 2022 (day 02), a total of 3.8 × 10^6^/kg CD34^+^ stem cells were infused. The patient received an infusion of CD19 CAR T cells (relmacabtagene autoleucel, relma-cel, JWCAR029, 2.7 ml containing 10^8^ CAR T cells) on June 1, 2022 (day 3). Platelets were 35×10^9^/L, implanted on June 18, 2022 (day 20); neutrophils were 1.52×10^9^/L implanted on June 19, 2022 (day 21); lymphocytes were 1.6×10^9^/L on July 7, 2022 (day 39). CD4 was 374/ul, IgG 5.57g/L, IgA 0.25g/L, IgM 0.12g/L on July 7, 2022 (day 39).

On the 4^th^ day after ASCT (the 1^st^ day after CAR T-cell infusion), the patient’s oral mucosa showed pseudomembranes and ulcers, and he had diarrhea 3-5 times a day but was able to eat and swallow liquid diet. Therefore, a diagnosis of mucositis grade 2 was made, which lasted for 6 days and gradually recovered. Additionally, on the 2^nd^ day after CAR T-cell infusion, the patient developed a fever with a temperature of approximately 38.5°C and was not effectively treated with nonsteroidal antipyretic analgesic drugs and antibiotics. Repeated bacterial blood cultures were negative. On the 4^th^ day after CAR T-cell infusion, the patient’s blood pressure was 88/49 mmHg (the IL-6 level was 659.7 pg/ml) and was not effectively treated with rapid fluid replenishment. The patient was diagnosed with CRS grade 2 and treated with one dose of 8 mg/kg tocilizumab. His blood pressure gradually returned to normal within a few hours, and his temperature gradually returned to normal within 2 days. There was no ICANS after CAR T-cell infusion.

**Figure 3:**
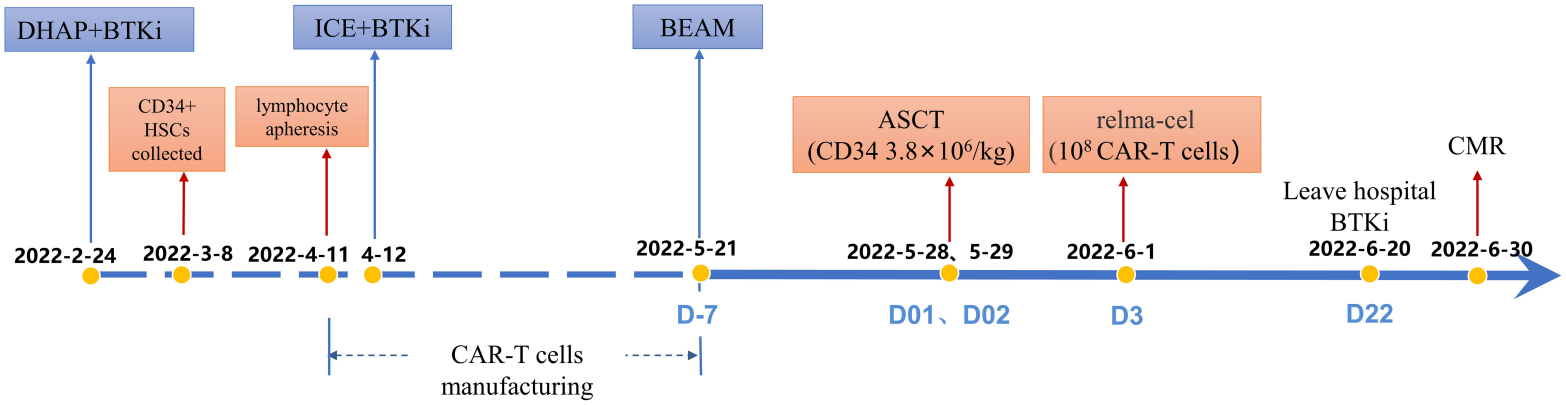
The timeline of ASCT combined with CAR T-cell therapy.

Surprisingly, PET-CT examination demonstrated CMR in the +1 month after CD19 CAR T-cell infusion following ASCT (**Figure 2B**). At the last follow-up, the patient received ibrutinib as maintenance therapy and remained in CMR (**Figure 2C**). Importantly, the circulating tumor DNA (ctDNA) results indicated that the mutations of *TP53*, *KMT2D*, *MS4A1*, *SOCS1* and *HIST1H1C* were persistently negative after CD19 CAR T-cell infusion following ASCT (**Table 2**). The timeline of treatment is shown in **Figure 3**.

**Table 2:** The ctDNA results after CD19 CAR T-cell infusion following ASCT.

Gene	Variant sites (HGVS)	2022.5.20 ctDNA	2022.5.27 ctDNA	2022.6.15 ctDNA	2022.6.29 ctDNA	2022.8.1 ctDNA	2022.9.1 ctDNA	2023.4.13 ctDNA
*HIST1H1C*	NM_005319(HIST1H1C):c.331G>A(p.A111T)	0.74%	–	–	–	–	–	–
*KMT2D*	NM_003482(KMT2D):c.12667C>T(p.Q4223X)	0.88%	–	–	–	–	–	–
*MS4A1*	NM_152866(MS4A1):c.160-1delG	1.22%	–	–	–	–	–	–
*SOCS1*	NM_003745(SOCS1):c.174C>G(p.F58L)	0.51%	–	–	–	–	–	–
*TP53*	NM_000546(TP53):c.713G>A(p.C238Y)	0.51%	–	–	–	–	–	–

% refers to the gene mutation frequency. (-), negative.

## Discussion

Histologic transformation of FL to DLBCL occurs in 10%–70% of patients over time, with a risk of 2% - 3% per year; this transformation is associated with an increased rate of mortality, especially in patients who progress early after immunochemotherapy (2, 18, 19). However, prospective randomized studies or clinical trials always exclude patients with transformed lymphoma, and there is no standard therapy strategy to guide practice in the modern era. The treatment choice is often individualized depending on the previous treatment history (2). For patients who are anthracycline naïve, R-CHOP or other anthracycline-based therapy approaches are suggested, which could yield a similar response to patients with *de novo* DLBCL. Patients with anthracycline exposure could benefit from salvage chemotherapy and consolidative ASCT. However, the role of ASCT in tFL patients with bendamustine exposure remains unclear (2). Hence, the optimal treatment strategies to overcome the poor prognosis of these transformed patients have yet to be determined.

In our present study, the patient received R-CHOP as initial therapy and BR and GB as salvage chemotherapy, but no effects were observed. NGS of the lymphoma tissue showed an *MS4A1* gene mutation that resulted in the loss of CD20 expression, which is most likely the reason that BR and GB failed (20). Moreover, the disease transformed to DLBCL with both anthracycline and bendamustine exposure, which poses challenges in selecting therapy approaches. First, in terms of clinical manifestations, the patient underwent POD24 and transformation early after the third line of therapies, with a bulky tumor mass and higher serum LDH level. All of these characteristics portended a poor prognosis (21). Second, in terms of pathological features, the disease transformed to DLBCL with double expression of Bcl-2 and c-myc and negative CD20 expression. The CD20 level was proven to be an independent factor of poor prognosis in newly diagnosed DLBCL patients (22). A study showed that for R/R DLBCL, 26.3% (5/19) of patients were confirmed to be CD20 negative according to posttreatment rebiopsy. The OS of all 5 patients was less than 11 months from CD20-negative transformation (23). The other study found that CD20 loss occurred in 16% of R/R FL patients, whose median OS was significantly shorter than that of CD20-positive patients (8.9 months vs. 28.3 months) (24). These results indicated that CD20 loss was related to a poor prognosis of B-NHL. Third, in genomic profiles, there were more than 10 gene mutations, including *TP53*, *KMT2D*, *MS4A1*, *CD83*, *DUSP2*, *EZH2*, *FOXO1*, *HIST1H1C*, *HIST1H1E*, *NFKBIA*, *SGK1*, *SOCS1*, *STAT3*, and *TBL1XR1*, when the disease underwent histologic transformation. Mutations in *TP53*, *KMT2D*, *HIST1H1C* and *TBL1XR1* always existed from onset to transformation. *TP53* confers lymphomas with poor outcomes, which cannot be overcome by chemotherapy or HSCT (5). Even in the era of cellular therapy, *TP53* mutations and/or copy number alterations were independent factors correlated with lower CR and shorter OS for R/R DLBCL patients treated with CD19 CAR T cells. The 1-year OS was 44% in *TP53*-altered patients and 76% in *TP53* wild-type patients (15). Therefore, the treatment strategy for our present case should be explored innovatively to improve the prognosis to the greatest extent.

For relapsed or refractory lymphomas, high-dose chemotherapy and ASCT (HDT-ASCT) following salvage chemotherapy is a common strategy, especially for those who achieve a partial remission (PR) after salvage chemotherapy (8). However, there are few therapeutic options for patients who are chemoresistant, resulting in lower response rates and shorter OS. In recent years, CAR T-cell therapy has emerged as a revolutionary treatment for R/R NHL to improve prognosis. CAR T cells are preferred as the first cellular immunotherapy with a lower nonrelapse mortality rate and similar relapse incidence, progression-free survival (PFS) and OS rates compared with allo-HCT for multiple R/R DLBCL (10). There have been three FDA-approved CAR T-cell products for R/R DLBCL or FL. For FL patients, the CR rates of CD19 CAR T-cell therapy were approximately 60%-65.4% (14, 25). The efficacy of CD19 CAR T-cell therapy for R/R DLBCL was inferior, with 44%-58% CR and 44%-65% 1-year PFS (11–13). Notably, for more than half of the enrolled patients with POD24, the CR rates (CRR) were 55% and 59% in the ZUMA-5 and ELARA trials, respectively (14, 25). Importantly, the JULIET, ZUMA-1 and TRANSCEND studies reported that patients with transformed follicular lymphoma achieved a notable duration of response. Compared with *de novo*/primary DLBCL, transformed/secondary DLBCL is associated with a more favorable outcome, including higher CRR, PFS, OS and lower mortality rates after CAR T-cell therapy (26). In the ZUMA-7 study, compared with standard care, axi-cel therapy led to significant improvements with higher response (83% vs. 50%) and CRR (65% vs. 32%), and it resulted in longer median event-free survival (EFS) in axi-cel therapy (8.3 months vs. 2.0 months), and the 24-month EFS was 41% and 16%, respectively (27). The real-world data suggested that the primary efficacy of CAR T-cell therapy in DLBCL with 32-66% CRR still has significant room for improvement (28). In addition, the durable remission rate is much lower at 30 to 40%. Furthermore, relapse after CAR T-cell therapy remains an important problem (28).

Recently, the efficacy of CAR T-cell therapy combined with HDT-ASCT has been confirmed in several clinical studies. Compared with CAR T cells alone, the ORR, CRR and long-term outcome were significantly improved after CAR T-cell infusion following ASCT therapy (5, 29). Compared with ASCT alone, significantly higher CRR and 3-year PFS rates and lower rates of 3-year relapse/progression were achieved after CAR T-cell infusion following ASCT (16). *TP53* genomic alterations have been shown to be associated with inferior CRR and OS rates in multivariable regression models among patients with LBCL treated with CD19 CAR T-cell therapy (15). However, combining CAR T cells with ASCT is effective for R/R aggressive B-NHL with *TP53* alterations, leading to long-term outcomes (5). Sandwich therapy of CAR T cells combined with ASCT could lead to long-term survival in patients with R/R Burkitt’s lymphoma with *TP53* mutations (30). An open-label single-arm prospective clinical study demonstrated that ASCT and sequential CD19/CD22 CAR T-cell cocktail therapy showed high CRR in R/R aggressive B-NHL, which was ineffective with chemotherapy (17). The safety of CAR T-cell therapy combined with ASCT was proven not only in case report studies but also in larger sample studies (5, 17, 30). In a single-arm study, grade 3 CRS occurred in only 2 patients (2/42), and grade 3 neurotoxicity occurred in 5% of patients (17). Compared with CAR19/22 T-cell cocktail treatment, CAR19/22 T-cell treatment in combination with ASCT reduced the occurrence of severe CRS (10.7% vs. 37.5%) and similar incidence rates of ICANS (9.1% vs. 19.3%) (5). These results indicated that the adverse events in these studies were manageable and reversible for most patients.

Relma-cel is a CD19-targeted, second-generation CAR T-cell product with a 4-1BB costimulatory domain manufactured in China. A multicenter trial conducted in China demonstrated its efficacy and safety in R/R DLBCL patients (31). Surprisingly, the patient received Relma-cel following ASCT and achieved CR at +1 month. There are several potential synergistic mechanisms of the CAR T cell-therapy combined with ASCT strategy. First, ASCT could eradicate lymphoma cells nonselectively to reduce tumor load. Second, myeloablative conditioning improves the bone marrow microenvironment by restraining immunosuppressive elements such as monocytes and macrophages (32). Third, the effect of ASCT on lymphodepletion is superior to that of traditional fludarabine and cyclophosphamide, and CAR T cells that are highly activated during hematopoietic reconstitution could further eliminate residual tumors (5, 33). The patient experienced grade 2 CRS and no ICANS, which indicates the good safety of this treatment strategy (34). The findings of the present case study suggest the high efficacy and good safety of combined ASCT and CAR T-cell therapies, which provide a novel approach for such patients. However, the follow-up time was relatively short, and long-term observation is needed to confirm the enduring efficacy of the combination therapy.

In some literature, the synergistic effect of ibrutinib and CAR-T cells has been studied, which indicated that the previous or concurrent ibrutinib treatment might overcome the resistance of CAR-T therapy (35, 36). Ibrutinib could effectively restore the numbers and function of T cells via inhibition of interleukin-2 inducible T cell kinase, which has a positive effect on anti-CD19/CD3 bispecific antibodies and CAR T cells (37–39). In chronic lymphocytic leukemia, ibrutinib increased the proportion of CAR T cells with less-differentiated naïve-like phenotype and also inhibited the expression of exhaustion markers to enhance CAR T cell function (40, 41). In R/R NHL or CLL, supplement of ibrutinib or acalabrutinib also improved the CAR T function, and ibrutinib causing the emergence of type 1 T-helper memory-like T-cell phenotype, which was not found with acalabrutinib (41, 42). Furthermore, ibrutinib could inhibit malignant B cells homing to spleens and lymph nodes through reducing homing chemokines CXCR4, which resulted tumor cells moving into the circulation and are killed by CAR T cells (43). The addition ibrutinib to CART for mantle cell lymphoma led to better response and longer survival in a xenograft mice model (44, 45). Take the above into consideration, we supplemented ibrutinib to combinatorial ASCT and CAR T therapies in our patient with a surprising efficacy.

In summary, we presented the first successful case of combination therapy with ASCT and CAR T cells for the treatment of relapsed and refractory CD20-negative tFL with *TP53* mutation and a bulky mass. Our study highlights the potential therapeutic strategy in tFL, which deserves further investigation in a large population in the future.

## Data availability statement

The original contributions presented in the study are included in the article/supplementary material. Further inquiries can be directed to the corresponding authors.

## Ethics statement

The studies involving humans were approved by The Ethics Committee of the First Affiliated Hospital of China Medical University. The studies were conducted in accordance with the local legislation and institutional requirements. The participants provided their written informed consent to participate in this study. Written informed consent was obtained from the individual(s) for the publication of any potentially identifiable images or data included in this article.

## Author contributions

JZ: Writing – original draft. DC: Writing – original draft. RG: Writing – review & editing. YM: Data curation, Writing – review & editing. YC: Data curation, Writing – review & editing. ZL: Investigation, Writing – review & editing. HZ: Investigation, Writing – review & editing. XY: Supervision, Writing – review & editing. NS: Supervision, Writing – review & editing.
